# Membrane-associated RING-CH protein (MARCH8) is a novel glycolysis repressor targeted by miR-32 in colorectal cancer

**DOI:** 10.1186/s12967-022-03608-z

**Published:** 2022-09-05

**Authors:** Zhan Wang, Miao-Miao Wang, Yan Geng, Chen-Yang Ye, Yuan-Sheng Zang

**Affiliations:** 1 Department of Medical Oncology, Second Affiliated Hospital of Naval Medical University, No. 415 Fengyang Road, Shanghai, 200003 China; 2 Department of Nursing, Zhabei Branch Hospital, Second Affiliated Hospital of Naval Medical University, No. 619, Zhonghuaxin Road, Shanghai, 200070 China

**Keywords:** Colorectal cancer, Membrane-associated RING-CH protein 8 (MARCH8), Glycolysis, HK2, miR-32

## Abstract

**Background:**

Colorectal cancer (CRC) is the third most common cancer and leading cause of cancer-related deaths worldwide. Aberrant cellular metabolism is a hallmark of cancer cells, and disturbed metabolism showed clinical significance in CRC. The membrane-associated RING-CH 8 (MARCH8) protein, the first MARCH E3 ligase, plays an oncogenic role and serves as a prognostic marker in multiple cancers, however, the role of MARCH8 in CRC is unclear. In the present study, we aimed to investigate the biomarkers and their underlying mechanism for CRC.

**Method:**

In this study, we first examined the function of MARCH8 in CRC by analysing public database. Besides, we performing gene silencing studies and generating cellular overexpression and xenograft models. Then its protein substrate was identified and validated. In addition, the expression of MARCH8 was investigated in tissue samples from CRC patients, and the molecular basis for decreased expression was analysed.

**Results:**

Systematic analysis reveals that MARCH8 is a beneficial prognostic marker in CRC. In CRC, MARCH8 exhibited tumor-suppressive activity both in vivo and in vitro. Furthermore, we found that MARCH8 is negatively correlated with hexokinase 2 (HK2) protein in CRC patients. MARCH8 regulates glycolysis and promotes ubiquitination-mediated proteasome degradation to reduces HK2 protein levels. Then HK2 inhibitor partially rescues the effect of MARCH8 knockdown in CRC**.** Poised chromatin and elevated miR-32 repressed MARCH8 expression.

**Conclusion:**

In summary, we propose that in CRC, poised chromatin and miR-32 decrease the expression of MARCH8, further bind and add ubiquitin, induce HK2 degradation, and finally repress glycolysis to promote tumor suppressors in CRC.

**Supplementary Information:**

The online version contains supplementary material available at 10.1186/s12967-022-03608-z.

## Introduction

Colorectal cancer (CRC) is the third most common cancer and leading cause of cancer-related deaths worldwide [1, 2]. In China, CRC is one of the most common tumours, and its incidence is gradually increasing [3]. Aberrant cellular metabolism is a hallmark sign of cancer cells [4], and disturbed metabolism is clinically significant in CRC [5–7]^.^ However, with advances in surgery, radiation, chemotherapy, immunotherapy, and targeted therapies, the five-year survival rate after diagnosis for metastatic CRC patients remains less than 20% [8, 9]. Therefore, identifying effective biomarkers and developing effective therapeutics for CRC is essential.

Membrane-associated RING-CH (MARCH) proteins belong to a newly identified subfamily of RING E3 ligases, and 11 MARCH proteins have been identified [10]^.^. Clinically, elevated MARCH8 levels have been observed in oesophageal tumours [11] and have been linked with poor prognosis in non-small-cell lung cancer [12]. Interestingly, MARCH8 was also reported to play an oncogenic role in gastric cancer [13, 14], oesophageal tumours [11, 15], and glioma [16]. Some of its functions have been studied; for example, Fan et al. indicated that microRNA-199a-5p regulates glioma progression by targeting MARCH8 [16]. Chen et al. revealed that MARCH8 suppresses tumour metastasis and mediates the degradation of STAT3 and CD44 in breast cancer cells [17]. Moreover, MARCH8 demonstrates tumour-suppressive activity in breast cancer [17] and is involved in cancer cell apoptosis in CRC [18]. However, the role of MARCH8 in CRC, its downstream targets, and the related mechanism of MARCH8 remain largely unexplored.

In this study, we aimed to investigate biomarkers and their underlying mechanism in CRC. First, the function of MARCH8 in CRC was studied using bioinformatics analysis, in vitro cellular assays, and in vivo xenograft models. Further results showed that MARCH8 regulates glycolysis. Then, the protein substrate in MARCH8 was identified and validated. Finally, the expression of MARCH8 in CRC samples was investigated, and the reason behind the decreased expression was analysed. We first linked the E3 ubiquitin ligase MARCH8 with glycolysis and then uncovered a related mechanism for MARCH8 in CRC.

## Materials and methods

### Study subjects

We collected 85 tumours and paired adjacent tissue from 85 CRC patients who were undergoing treatment at Shanghai Changzheng Hospital, Shanghai, China from January 2007 to December 2008. To avoid interference by other treatment methods, we restricted our study to include patients who only underwent surgical resection and excluded those treated with other methods. Clinicopathological information, including the patient's sex, age, tumour size, and tumour stage, was summarized from the medical records of the patients (Additional file 1: Table S1). All the protocols used in this study followed the Declaration of Helsinki and were approved by the Ethical Review Board of Shanghai Changzheng Hospital. Twenty-five tumors with paired normal tissue of the 85 patients were immediately frozen in liquid nitrogen and stored at − 80 °C until subsequent analysis. We also obtained paraffin-embedded tumor tissues from all 85 patients.

### Data collection and processing

The expression level, survival prognosis, and clinical characteristics information of MARCH1-11 proteins was downloaded through public databases TCGA (https://www.cancer.gov/about-nci/organization/ccg/research/structural-genomics/tcga) [19], and GEO public database (https://www.ncbi.nlm.nih.gov/). The TCGA-COAD included 571 samples and READ-TCGA included 192 samples; GSE39582 contained 443 colon cancer and 19 non-tumoral colorectal mucosas. GSE41258 contained 390 expression arrays from primary colon adenocarcinomas, adenomas, metastasis, and corresponding normal mucosae. GSE12945 included microarray data of 62 CRC patients. GSE17537 and GSE17536 included 55 colorectal cancer patients from Vanderbilt Medical Center (VMC) were used as the training dataset and 177 patients from the Moffitt Cancer Center were used as the independent dataset. GSE14333 contained samples taken from colorectal cancers in surgically resected specimens in 290 colorectal cancer patients. The expression level Boxplot was analysed by ggplot2 R package, the forest R package was used for forest diagram, and survminer R package was carried for survival analysis.

### Immunohistochemistry (IHC) analysis

Paraffin-embedded tumour tissue sections from the 85 patients were subjected to IHC analysis. We counted the number of MARCH8-positive cells in five random visual fields under a 40 × microscope, and then calculated an average value of 20% to be the cut-off value. All 85 patients were divided into two groups based on the percentage of MARCH8-positive cells. First, the sections were deparaffinized with xylene and rehydrated in graded alcohol, i.e., 100, 95, 80, and 70% ethanol. After rehydration, we immersed the sections in a 0.01 M citrate buffer (pH 6.0) and heated the samples in a microwave oven for 15 min at 850 W to retrieve the antigen. We then incubated the sections for 30 min in 0.3% hydrogen peroxide and then in a 10% heat-inactivated goat serum. After an incubation with anti-MARCH8 antibody (ab109690, Abcam, USA) at 4 °C overnight, the sections were probed with a horseradish peroxidase-conjugated secondary antibody for one hour at 25 °C. Finally, MARCH8-expressing cells in the tumour sections were visualized with a 3,3-diaminobenzidine solution, and the sections were counterstained for 3 min with haematoxylin.

### Cell culture

HEK293T, HIEC-6, CACO2, HT29, HCT116, LOVO, RKO, and SW480 cells were acquired from the Shanghai Cell Bank, Chinese Academy of Sciences, China. The cell lines mentioned above were maintained in Dulbecco's modified Eagle’s medium (DMEM, HyClone, USA) supplemented with 10% foetal bovine serum (Gibco, USA), 100 units/mL penicillin, and 100 μg/mL streptomycin in a 37 °C humidified cell culture incubator with 5% CO_2_.

### Construction of lentiviral plasmids and lentivirus packaging

The *MARCH8* coding sequence was synthesized by Shanghai Sangon Biotech Co. Ltd. We cloned the *MARCH8* coding sequence into the pLVX-puro vector (Clontech, USA) to construct a lentiviral *MARCH8*-overexpressing plasmid to overexpress *MARCH8* in CRC cells. We synthesized three short hairpin RNAs (shRNAs) targeting human *MARCH8* (sequences shown in Additional file 1: Table S2) and cloned them into the pLKO.1 vector (Addgene, USA) to silence endogenous *MARCH8* in CRC cells. We also synthesized a negative control shNC and cloned it into the pLKO.1 vector. For the lentivirus packaging, each constructed plasmid was transfected into HEK293T cells and packaging plasmids psPAX2 and pMD2.G using the Lipofectamine 2000 cell transfection kit (Invitrogen, Carlsbad, USA).

## Lentiviral infection

CACO2 and SW480 cells were infected with lentivirus to produce oeMAR cells or vector cells, respectively. LOVO cells were infected with lentivirus expressing shMAR or shNC to produce shMAR LOVO cells and shNC LOVO cells, respectively. We treated shMAR-1 LOVO cells and shNC LOVO cells with 0.5 mM lonidamine (Selleck, Houston, USA) [20] or with the same volume of DMSO for 24 h to investigate whether MARCH8 has a specific inhibitory effect on HK2. CACO2 and SW480 cells showed low endogenous basal expression and were chosen for MARCH8 overexpression (oeMAR cells).

### Real-time polymerase chain reaction (RT–PCR)

We extracted total RNA using TRIzol reagent (Invitrogen, Carlsbad, USA) following the manufacturer’s instructions. The isolated total RNA was then subjected to first-strand cDNA synthesis using the Reverse Transcription Kit (Thermo Fisher, USA). The SYBR Green Mix Kit (Thermo Fisher, USA) was used to run real-time PCR on an ABI 7500 Real-time Platform (Applied Biosystems, USA). Relative mRNA expression was calculated based on the 2^−ΔΔCt^ method. Human *GAPDH* was used as the internal reference gene. The primers are shown in Additional file 1: Table S3.

### Western blotting

Tissue samples (approximately 0.2 g) were powdered in liquid nitrogen and then resuspended in a RIPA buffer supplemented with protease inhibitor cocktail (Sigma, USA). Cell lysates were prepared by resuspending cell pellets in RIPA buffer supplemented with protease inhibitor cocktail. After blocking with 5% skim milk, the membranes were probed with MARCH8 and metabolism gene primary antibodies at 4 °C overnight. After washing away unbound antibodies with tris-buffered saline and 0.1% Tween® 20 (TBST), the membranes were incubated at 25 °C for one hour with HRP-conjugated rabbit secondary antibody (Beyotime, China). Finally, an enhanced chemiluminescence (ECL) system (Millipore, USA) was utilized to visualize the protein bands.

SW480 and CACO2 cells were lysed with a mixed buffer that contained RIPA, NaF, and PMSF (Beyotime, China) on ice for 30 min. Then the collected protein was denatured in a 95 °C water bath for 10 min and centrifuged at 12,000 rpm at 4 °C for 10 min. The upper clear cell lysates were transferred to new tubes. Equal amounts of protein were loaded on gels and separated by SDS–PAGE. Then, proteins were transferred to PVDF membranes (Immobilon-P; Millipore, Billerica, Massachusetts) and blocked with 5% milk or bovine serum albumin, followed by incubation with primary and secondary antibodies. Primary antibodies were anti-CDK4 (66,950-1-Ig, Proteintech, 1:1000), cycling D1 (60186-1-Ig, Proteintech, 1:20000), cleaved caspase 3 (19677-1-AP, Proteintech, 1:500) and β-actin (66009-1-Ig, Proteintech, 1:10000) antibodies.

### Coimmunoprecipitation (Co-IP)

For Co-IP analysis, cell lysates were incubated with anti-MARCH8 antibody (Abcam, USA), anti-HK2 antibody (Abcam, USA), or IgG (Abcam, USA) for one hour at 4 °C. After that, protein A/G-agarose beads were added to the lysates, and the mixture was incubated for three hours at 4 °C to pull down the antigen–antibody complexes. Finally, the precipitates were eluted with elution buffer and detected by Western blot analysis.

### Cell proliferation assay

We utilized the Cell Counting Kit-8 (CCK-8, Dojindo Laboratories, Japan) to evaluate the proliferation of CRC cells. Briefly, 2 × 10^3^ cells were seeded into each well of 96-well plates. Then, 10 μL CCK-8 reagent was added to each well, and the mixtures were incubated at 37 °C for an additional hour. The number of viable cells was then calculated based on the optical absorbance values measured at 450 nm using the Multiskan MS Plate Reader (Labsystems, Finland).

### Determination of the oxygen consumption rate (OCR) and extracellular acidification rate (ECAR)

We measured the OCR and ECAR using a Seahorse XF24 Extracellular Flux Analyser (Seahorse Bioscience, USA). Briefly, 1 × 10^5^ CRC cells were seeded in each well of XF24 Cell Culture Microplates (Seahorse Bioscience, USA). One hour before the assay, we replaced DMEM with XF Assay Medium (Seahorse Bioscience, USA) (pH 7.4). For OCR measurements, 2 mM oligomycin, 3 mM carbonyl cyanide-4 (trifluoromethoxy) phenylhydrazone (FCCP), and a mixture of 2 mM rotenone and 5 mM antimycin were sequentially added to the reaction system to determine the basal respiration. For ECAR measurements, 5.5 mM glucose, 2 mM oligomycin, and 100 mM 2-deoxyglucose (2-DG) were added sequentially to the reaction system to determine the glycolytic rate, glycolytic reserve, and nonglycolytic ECAR.

### Nude mice xenograft model

All animal experiments in this study were performed under the supervision of the Animal Care Committee of Shanghai Changzheng Hospital. Ten four-week-old male BALB/c nude mice (SLRC Laboratory Animal, China) were randomized into two groups (n = 5 per group) and subcutaneously injected with 5 × 10^6^ oeMAR SW480 cells (the oeMAR group) or vector SW480 cells (the vector group). To evaluate the degree of apoptosis in the tumour tissues, a TUNEL assay kit (Roche, USA) was used. The tumour volume in each mouse was determined every three days for the next 33 days according to the following equation: tumour volume = 1/2 × (the largest diameter) × (the smallest diameter) ^2^. The mice were euthanized after 33 days, and the volumes and wet weights of tumour tissues were determined.

Twenty-four-week-old male nude BALB/c mice were randomized into four groups (n = 5 per group) to investigate whether MARCH8 has a specific inhibitory effect on the functions of HK2 in CRC cells in vivo. The vehicle-shMAR group was subcutaneously injected with 5 × 10^6^ shMAR-1 LOVO cells, and the vehicle-shNC group was subcutaneously injected with 5 × 10^6^ shNC LOVO cells. Fifteen days later, the mice in the above two groups were intraperitoneally injected with DMSO twice a week. The Lonidanmine-siNC group was subcutaneously injected with 5 × 10^6^ shNC LOVO cells and intraperitoneally injected with a lonidamine-DMSO solution at a dose of 100 mg lonidamine/kg body weight (twice a week) fifteen days after LOVO cell injection. The Lonidanmine-siMARCH8 group was injected with 5 × 10^6^ shMAR-1 LOVO cells subcutaneously and intraperitoneally with a lonidamine-DMSO solution at a dose of 100 mg lonidamine/kg body weight (twice a week) fifteen days after LOVO cell injection. The tumour volume in each mouse was then determined every three days using the method described above after the cells were injected.

### Luciferase reporter assay

The amplified MARCH8 3’-UTR-WT and MARCH8 3’-UTR-MUT were cloned into the pGL3 luciferase vector (Promega Corporation). CRC cells were seeded onto 96-well plates over a 24-h period. CRC cells were transfected with MARCH8 3’-UTR-WT or MARCH8 3’-UTR-MUT and miR-32 inhibitor using Lipofectamine 3000. After 48 h, cells were harvested, and the Dual Luciferase Reporter Assay Kit (Promega Corporation) was used to measure luciferase activity.

### Statistical analysis

We used GraphPad Prism software version 6.0 to analyse our data. We used Student’s *t test* and analysis of variance (ANOVA) to compare data from different groups. Pearson correlation analysis was applied to establish a linear correlation between MARCH8 and HK2. *P* values less than 0.05 were considered statistically significant.

## Results

### Systematic analysis revealed that MARCH8 is a beneficial prognostic marker in CRC

First, we analysed the expression, survival prognosis, and clinical characteristics of 11 MARCH proteins through public databases TCGA, GEO public databases. As shown in the results, MARCH8 presented lower expression level in CRC tissue compared to normal tissue (Additional file 1: Figs S1, S2). Besides, in general, MARCH8 plays a protective role in CRC (Hazard Ratio < 1). Considering its lower expression level and protective role in CRC, we chosen the MARCH8 for further investigation. Then, immunohistochemistry (IHC) was performed to examine the expression of MARCH8 proteins in tumour tissues. Patients with samples containing more than 20% MARCH8-positive cells (middle panel of Additional file 1: Fig. S3A) were classified into the MARCH8-high group, and those with less than 20% MARCH8-positive cells (right panel in Additional file 1: Fig. S3A) were classified into the MARCH8-low group. We examined *MARCH8* mRNA expression in 25 tumours with paired normal tissues stored at − 80 °C. *MARCH8* mRNA exhibited significantly decreased expression in the tumour samples, which is consistent with the previous results based on public datasets (Additional file 1: Fig. S3B). Kaplan–Meier analysis indicated that the five-year survival rate of the 34 patients in the MARCH8-high group was significantly higher than that of the 51 patients in the MARCH8-low group (*P* = 0.007) (Additional file 1: Fig. S3C). Last, chi-square test analysis for MARCH8 protein expression with clinicopathological parameters showed a significant correlation of MARCH8 protein with tumour size and TNM stage (Additional file 1: Table S4). And clinical characteristics of MARCH8 and other MARCH proteins by large samples of public databases TCGA (TCGA- COAD, TCGA-READ), GEO (GSE41258, GSE39582, and GSE12945) were also analysed (Additional file 2: Table S5 and Additional file 3: Table S6).

### MARCH8 acts as a tumour suppressor in CRC

**Fig. 1:**
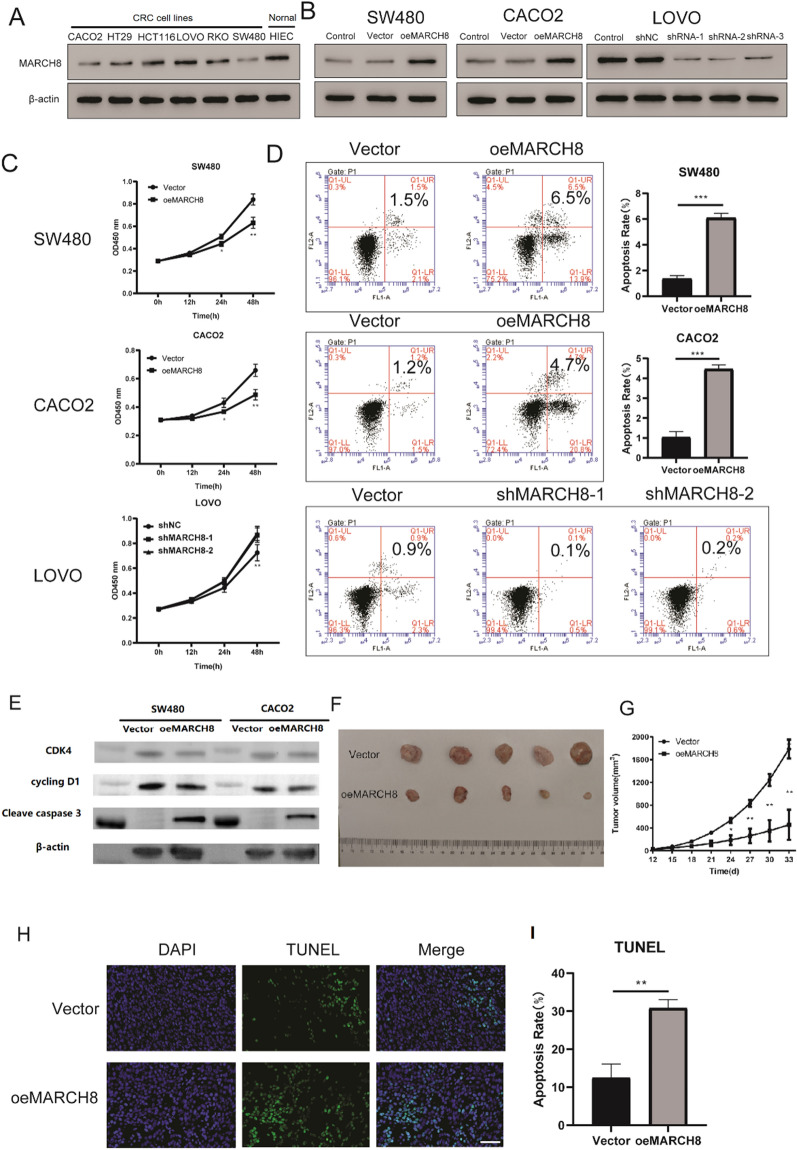
MARCH8 inhibits colorectal cancer (CRC) in vitro and in vivo. **A** MARCH8 expression in colorectal cells was examined. **B** Expression of MARCH8 in MARCH8-manipulated cells.** C** Proliferation curves and (**D**) apoptosis rates of MARCH8-manipulated CRC cells. **E** Tumour size in tumour tissues from the oeMAR mice and the vector mice. **F** Tumour growth curves of the oeMAR mice and the vector mice. **G** Quantification of apoptosis cell rates, as revealed by TUNEL assays, scale bar 50 μm. **H** Percentages of apoptotic cells in tumour tissues revealed by TUNEL assays. *P < 0.05, **P < 0.01, ***P < 0.001 vs. vector

We first examined the expression of MARCH8 in multiple cell lines (Fig. 1A) to investigate the function of MARCH8 in CRC cells. The expression of MARCH8 in LOVO cells was high, and LOVO cells were used for MARCH8 knockdown (shMAR). MARCH8 overexpression significantly enhanced MARCH8 abundance in CACO2 and SW480 cells, as shown in Fig. 1B. Three short hairpin RNAs (shMARs) targeting MARCH8 were designed to rule out off-target effects in LOVO cells. Of the three, shMAR-1 and shMAR-2 showed better inhibitory effects (Fig. 1B) and were used for the following phenotype assays. As shown in Fig. 1C, compared to that of other cells, CACO2 and SW480 oeMAR cells showed significantly decreased proliferation, and LOVO shMAR cells exhibited increased cell numbers, suggesting that MARCH8 may inhibit cell proliferation in CRC. Consistently, CACO2 and SW480 oeMAR cells showed increased apoptosis rates, and LOVO shMAR cells demonstrated slightly reduced apoptosis rates (Fig. 1D), implying that MARCH8 may also induce cell apoptosis. Furthermore, as seen in Fig. 1E, the WB results showed that the proliferation related markers CDK4 and cyclin D1 were decreased while the MARCH8 was overexpression. The apoptosis-related proteins caspase3 was significantly increase in the MARCH8 overexpression group, which was consistent with the previous results. Together, these results indicated that the MRCHA8 was a tumor suppressor in CRC in vitro.

We generated xenograft mouse models by subcutaneously injecting SW480 cells into nude BALB/c mice to verify the in vitro effect of MARCH8. As shown in Fig. 1F, G, tumour tissues in the oeMAR group grew significantly slower than those in the vector group. Tumour tissues were then subjected to a TUNEL assay to study the impact of MARCH8 on cell apoptosis. As shown in Fig. 1H–I, the ratio of apoptotic cells in the oeMAR group was significantly higher than that in the control group. Collectively, the in vitro and in vivo results suggested that MARCH8 is a tumour suppressor in CRC.

### MARCH8 inhibits glycolysis by ubiquitination-mediated HK2 degradation

**Fig. 2:**
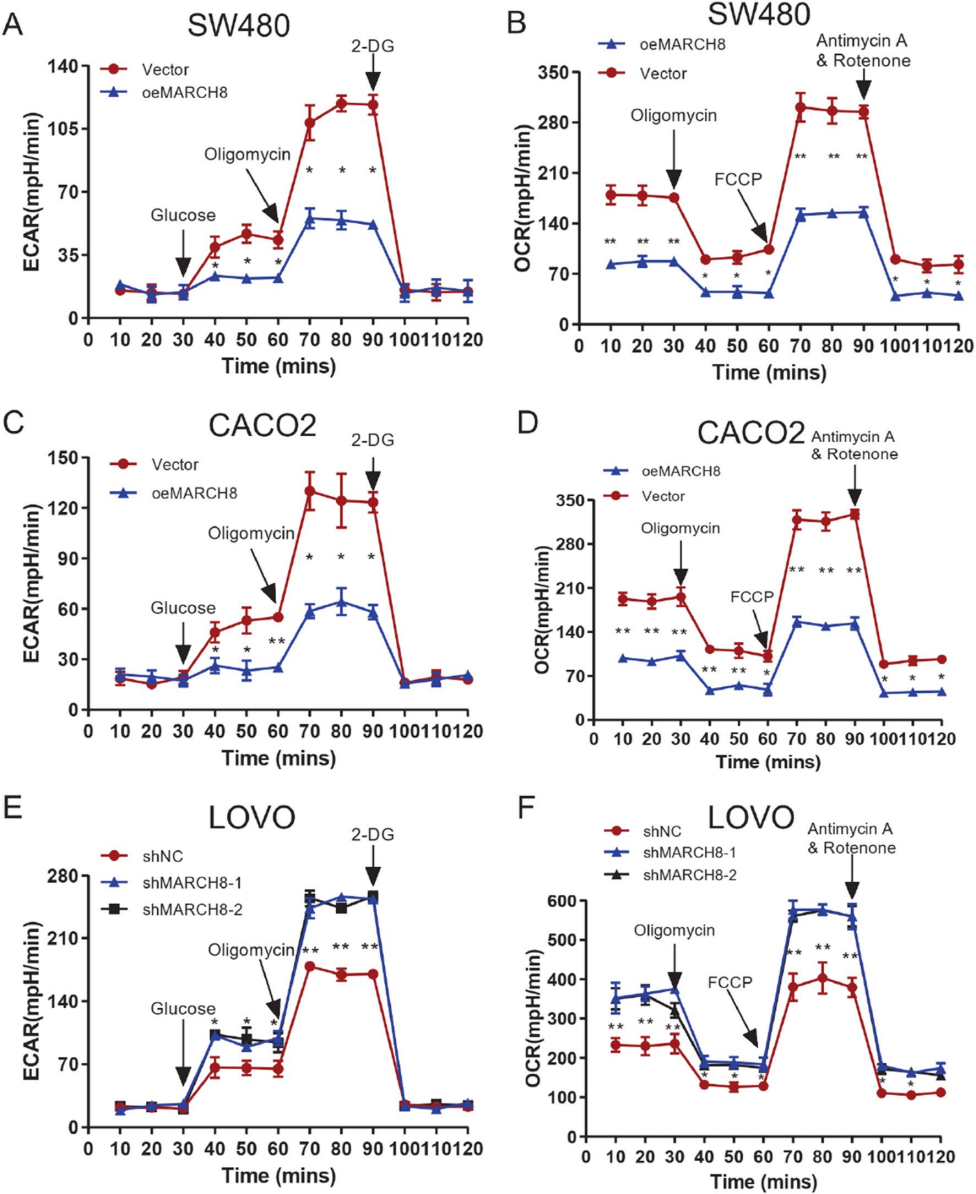
Role of MARCH8 in glycolysis and respiration in CRC cell lines. The ECAR and OCR results of SW480 oeMARCH8, CACO2 oeMARCH8 and LOVO shRNA cells are shown in (**A**, **B**) (SW480), (**C**, **D**) (CACO2) and (**E**, **F**) (LOVO), respectively. Each experiment was repeated at least three times. *P < 0.05, **P < 0.01 vs. vector

**Fig. 3:**
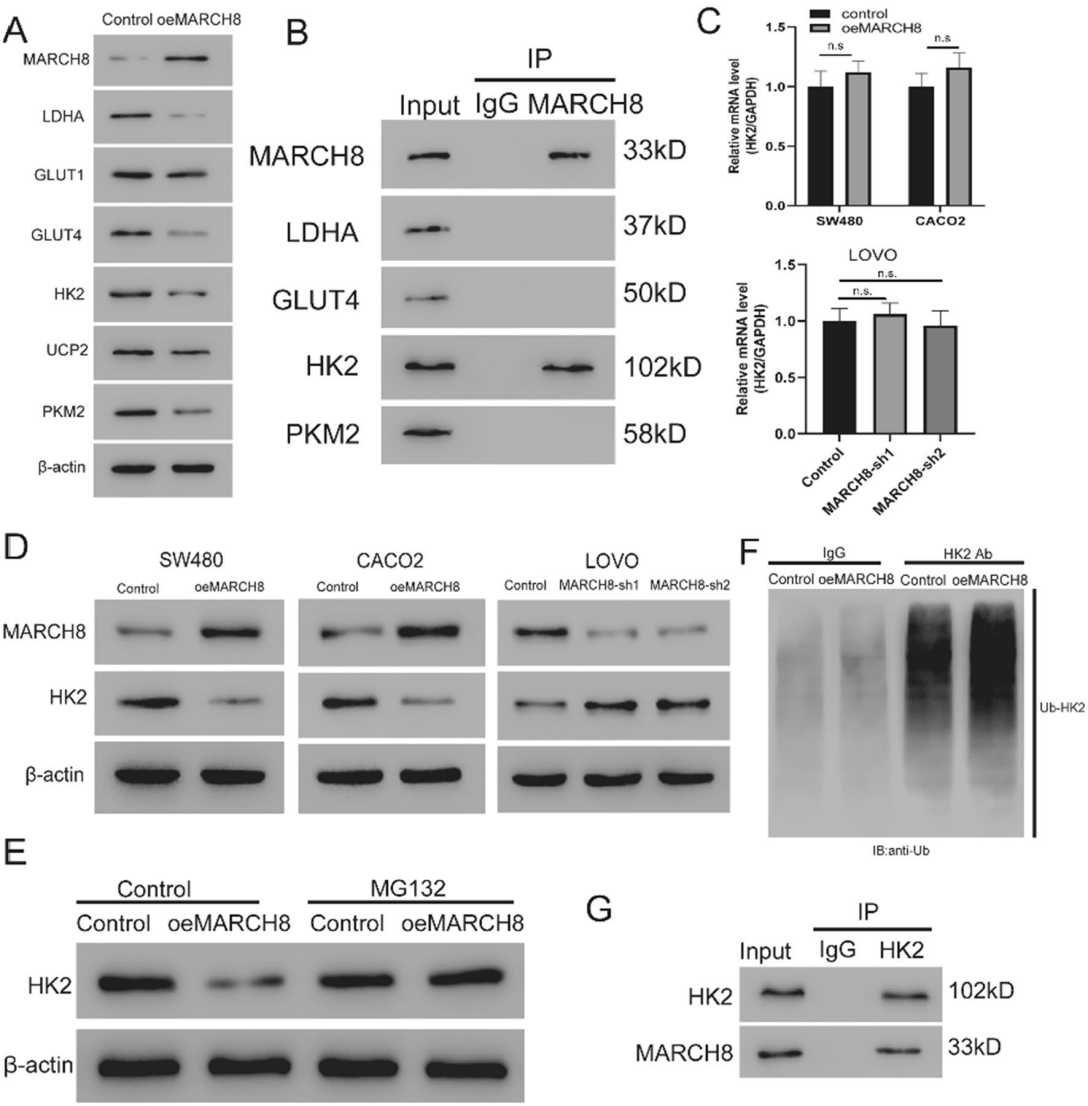
MARCH8 regulates the protein level of HK2 by promoting its ubiquitination in CRC cells. **A** Protein expression of glycolysis enzymes was examined in oeMARCH8 cells. **B** Immunoprecipitation bands showing the binding of MARCH8 with HK2. **C** Relative mRNA and (**D**) Protein expression levels of the HK2 gene in SW480 oeMARCH8, CACO2 oeMARCH8 and LOVO shRNA cells; n.s. indicated not significant. **E** Protein expression of HK2 in oeMAR cells with or without MG132 treatment; **F** Analysis of HK2 ubiquitination in oeMAR SW480 and vector SW480 cells; **G** Co-IP analysis of the interaction between MARCH8 and HK2 in SW480 cells Each experiment was repeated at least three times

Studies have shown that E3 ubiquitin ligases regulate glycolysis in CRC [21, 22], but no studies could be found regarding MARCH8 in cancer cell glycolysis. Therefore, the basal cellular respiration and maximal respiration rate were examined with ECAR and OCR assays to explore the role of MARCH8 in glycolysis. Figure 2A–D shows that MARCH8 overexpression inhibited glycolysis and respiration in CACO2 and SW480 cells. In LOVO cells, MARCH8 knockdown with shMAR-1 and shMAR-2 significantly increased the glycolytic rate and basal cellular respiration level (Fig. 2E, F). The phenotype of MARCH8-mediated inhibition of glycolysis prompted us to explore its mechanism in CRC. The expression of canonical glycolysis enzymes [23] was examined in oeMAR cells, including lactate dehydrogenase A (LDHA), glucose transporter 1 (GLUT1), glucose transporter 4 (GLUT4), hexokinase 2 (HK2), uncoupling protein 2 (UCP2), and pyruvate kinase M2 (PKM2). As shown in Fig. 3A, the protein levels of LDHA, GLUT4, HK2, and PKM2 were decreased upon *MARCH8* overexpression. Given that MARCH8 mediates substrate protein ubiquitination and degradation [14, 24], we next investigated the binding of MARCH8 with LDHA, GLUT4, HK2, and PKM2. Immunoprecipitation experiments showed that MARCH8 only interacted with HK2 (Fig. 3B). Therefore, we sought to investigate how MARCH8 regulates the HK2 protein. *MARCH8* overexpression resulted in decreased HK2 protein levels without affecting the transcription of *HK2* mRNA in CACO2 and SW480 cell lines (Fig. 3C, D). Consistent with these results, *MARCH8* knockdown led to elevated HK2 protein levels without affecting the transcription of *HK2* in LOVO cells (Fig. 3C, D). Interestingly, treating oeMAR SW480 cells with MG132, a potent proteasome inhibitor [25], offset the effect of *MARCH8* overexpression on the HK2 protein (Fig. 3E), indicating that MARCH8 regulates the expression of HK2 in a proteasome-dependent manner. Therefore, we investigated the effect of *MARCH8* on HK2 ubiquitination levels in SW480 cells. As shown in Fig. 3F, we indeed observed a higher HK2 ubiquitination level in oeMAR SW480 cells than in other cells. Finally, Co-IP was conducted to validate the MARCH8-HK2 binding complex (Fig. 3G). Together, these results demonstrate that MARCH8 promoted ubiquitination-mediated proteasome degradation to reduce HK2 protein expression.

### HK2 inhibitor partially rescues the effect of MARCH8 knockdown in CRC

**Fig. 4:**
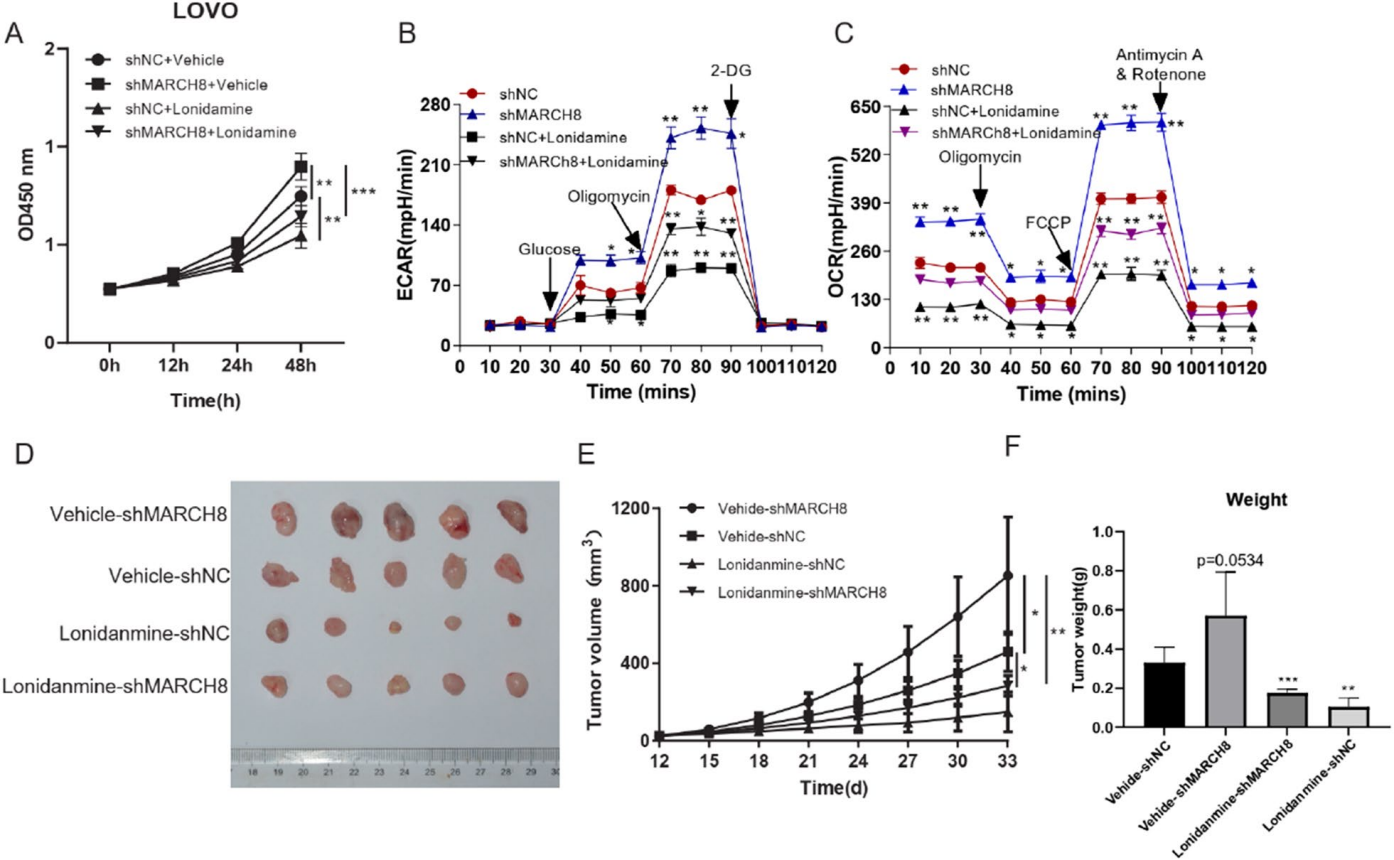
HK2 inhibitor partially rescues the effect of MARCH8 knockdown in CRC. **A** Proliferation curve of LOVO cells treated with shMARCH8 and lonidamine; **B** ECAR and (**C**) OCR curve of LOVO cells transfected with shMARCH8 and the HK2 inhibitor lonidamine **D** Tumour size of LOVO cells treated with shMARCH8 and the HK2 inhibitor lonidamine; **E** Tumour growth curve of LOVO cells treated with shMARCH8 and the HK2 inhibitor lonidamine; **F** Tumour weight of LOVO cells treated with shMARCH8 and the HK2 inhibitor lonidamine; *P < 0.05, **P < 0.01, ***P < 0.001 vs. vector

ShMAR-1 LOVO and shNC LOVO cells were treated with the HK2 inhibitor lonidamine to determine whether HK2 mediated the functions of MARCH8 in CRC. As shown in Fig. 4A–C, the effects of *MARCH8* knockdown on LOVO cell proliferation, glycolysis, and respiration were blocked by lonidamine treatment. Next, LOVO xenograft mouse models were constructed and treated with vehicle or lonidamine. Tumour tissues in the shMAR-1 + vehicle mice grew significantly faster than those in the shNC + vehicle mice. The growth rates of shMAR-1 + LND tumour tissues were significantly lower than those of shMAR-1 + vehicle tumour tissues (Fig. 4D–F).

### MARCH8 is negatively correlated with HK2 protein in CRC patients

Next, the correlation of MARCH8 with HK2 protein in CRC patients was analysed. We selected 8 patients and determined the protein levels of MARCH8 and HK2. CRC tumour samples had significantly lower MARCH8 levels (*P* < 0.0001) and significantly higher HK2 levels (*P* < 0.05) than those in normal colon tissues (Fig. Additional file 1: S4A, B). Pearson correlation analysis revealed that there is a significant negative correlation between MARCH8 and HK2 in CRC tumour tissues (*R* = -− 5672, *P* < 0.05) (Additional file 1: Fig. S4C). These results demonstrate that the protein level of MARCH8 is negatively correlated with that of HK2 in human CRC tumour tissues.

### Increased miR-32 attenuates MARCH8 expression in CRC

**Fig. 5:**
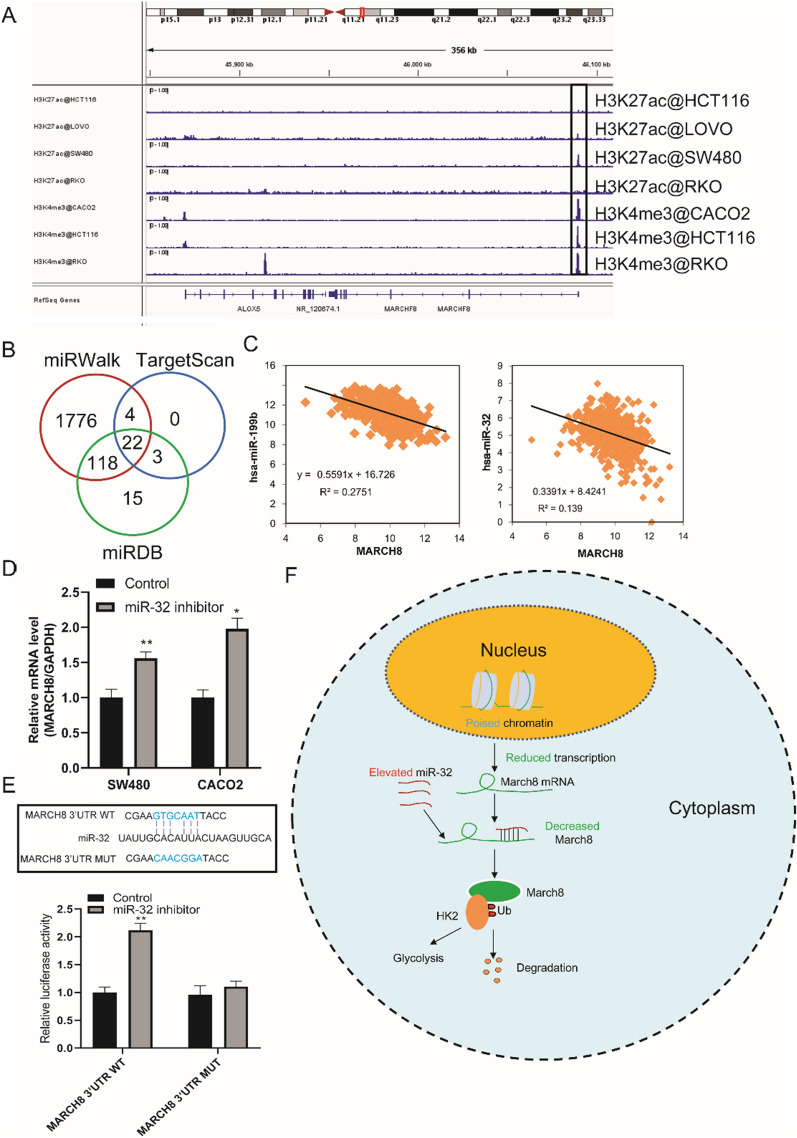
Poised chromatin and miR-32 decreased the expression of MARCH8 in CRC. **A** ChIP-Seq binding peaks in the MARCH8 gene promoter of histone modification marks; **B** MiRwalk, TargetScan and miRDB were applied to predict the binding sites of miRNAs in the MARCH8 3’UTR; **C** Expression correlation analysis of miR-199b and miR-32 with MARCH8 in TCGA COAD and READ datasets; **D** Expression of MARCH8 in colorectal cells was examined with qRT–PCR in miR-32 inhibitor-treated cells; **E** A luciferase assay was conducted to explore the binding sites of miR-32 with MARCH8 3’UTR sites. **F** Schematic figure showing the molecular mechanism of the miR-32/MARCH8/HK2 axis in CRC. *P < 0.05, **P < 0.01 vs. Vector

Finally, the molecular mechanism underlying the decreased expression of MARCH8 in CRC was investigated. First, we investigated the histone activation marks of MARCH8 in CRC cells. As shown in Fig. 5A, weak or no H3K27ac peaks were found in the promoter regions of HCT116, LOVO, SW480, and RKO cells, suggesting that chromatin may be in a poised state and that the transcription process is controlled. We then investigated the role of miRNAs, which are posttranscriptional regulators, in the regulation of MARCH8. Three online tools (MiRwalk, TargetScan, and miRDB) were used to predict the binding of miRNA to sequences in MARCH8 3’UTR regions, and all 22 common miRNAs were acquired as a result (Fig. 5B). The expression of these miRNAs and MARCH8 in CRC samples was then analysed, and two miRNAs, miR-199b and miR-32, showed a significant negative correlation with MARCH8 (Fig. 5C). Because miR-32 acts as an oncogene and unfavourable prognostic marker in CRC [26–30] while miR-199b plays the opposite role [31, 32], we chose miR-32 for further validation studies. Then, we transfected miR-32 inhibitors into CRC cells, and as shown in Fig. 5D, MARCH8 expression was significantly increased. Finally, a dual luciferase assay was conducted to demonstrate the binding of miR-32 to the MARCH8 3’UTR sequence. The MARCH8 3’UTR wild-type (WT) group showed increased luciferase activity, while no significant changes were observed in the mutant (MUT) group (Fig. 5E). In summary, in CRC, poised chromatin and miR-32 decreases the expression of MARCH8, which induces HK2 degradation, represses glycolysis and acts as a tumour suppressor in CRC (Fig. 5F).

## Discussion

MARCH8, the first MARCH E3 ligase to be identified [33], plays important roles in the immune response [11–13, 24, 34, 35]^.^ Controversial studies have been reported on the functions of MARCH8 during tumorigenesis. In a previous study, MARCH8 overexpression inhibited cell proliferation and increased cell apoptosis [35]. In another study, *MARCH8* knockdown impaired oesophageal squamous cell carcinoma cell proliferation but promoted cell apoptosis [11]. MARCH8 was also observed to have oncogenic roles in gastric cancer [36]^.^ In this study, we first demonstrated that MARCH8 inhibited tumorigenesis in vitro and in vivo.

Normal cells mainly rely on oxidative phosphorylation (OXPHOS) to supply energy. Unlike normal cells, ATP production in cancer cells is more dependent on glycolysis [37]. Glycolysis in HER2-positive tumour cells is regulated by the SKP2-SCF E3 ubiquitin ligase [38]. In addition, aerobic glycolysis in ovarian carcinoma is suppressed by CHIP (carboxyl terminus of HSC70-interacting protein) [39]. In the current study, *MARCH8* overexpression inhibited cell proliferation by impairing glycolysis and respiration. In contrast, *MARCH8* knockdown accelerated cell proliferation by promoting glycolysis and respiration in a CRC cell line with high basal MARCH8 expression.

It has been reported that MARCH8 possesses E3 ligase activity to promote the ubiquitination and endocytosis of the transferrin receptor [40, 41]. Here, we found that MARCH8 interacted with HK2. Previous studies have reported that E3 ubiquitin ligases promote HK2 ubiquitination [42]. Our data suggested that MARCH8 reduced HK2 protein levels by promoting its ubiquitination in CRC cells. Furthermore, a negative correlation between MARCH8 and HK2 protein levels was observed in clinical samples, which confirmed the in vitro findings. Treating CRC cells with the HK2 inhibitor lonidamine blocked the promotion of *MARCH8* knockdown in the proliferation and glycolysis of CRC cells. HK2 can maintain mitochondrial outer membrane integrity and prevent apoptosis [43]. We consistently observed that the antiapoptotic role of MARCH8 knockdown was reversed by lonidamine in xenograft experiments. Collectively, these findings indicate that MARCH8 can inhibit proliferation and glycolysis in CRC cells by down-regulating the protein level of HK2.

Finally, we investigated the mechanism of decreased MARCH8 expression in CRC. Weak transcriptional activation histone marks suggest that the expression of MARCH8 may be partially silenced at the transcriptional level. Moreover, we also explored the role of miRNAs in MARCH8 expression regulation. Increased miRNA expression is known to be a tumour-promoting molecular change that is due to the expression of tumour suppressors being repressed [44]. Here, we first demonstrated that miR-32 plays a pivotal role in decreased expression of MARCH8, establishing the miR-32/MARCH8/HK2 axis in CRC pathogenesis and progression.

In conclusion, our study elucidates the molecular mechanism underlying the inhibitory effect of MARCH8 on glycolysis in CRC. Our work establishes a novel regulatory miR-32/MARCH8/HK2 axis in the pathogenesis and progression of CRC. However, there are still some limitations in this work. For example, we should add the xenograft mouse models in the shMARCH8 group, and detect the expression of downstream targets HK2, promotion and apoptosis related proteins in the tubes of xenograft mouse models in the shMARCH8 group and oeMARCH8 group by immunohistochemistry. What’s more, the specific role of the miR-32/MARCH8/HK2 regulatory axis and its association with other regulatory factors should be explored. Further, more CRC patients’ samples should be collected for validation and the impact of other MARCH proteins on CRC should be conduct in-depth research. It should be noticed that these experiments would be performed in the future research.

## Supplementary Information

**Additional file 1: Figure S1.** The protein levels and Hazard Ratio of MARCH1-11 in TCGA-COAD, TCGA-READ, GSE12945 and GSE39582 datasets. **A** The protein levels of MARCH1-11 in TCGA-COAD, TCGA-READ, and GSE39582 datasets. **B** The forestplots of MARCH1-11 in TCGA-READ, GSE12945 and GSE39582 datasets. **Figure S2.** The expression levels and overall survival of MARCH8 in public datasets. **A** The expression levels of MARCH8 in TCGA-COAD, TCGA-READ, GSE41258 and GSE39582 datasets. **B** The overall survival of MARCH8 in TCGA-COAD, GSE41258, GSE12945, GSE39582, GSE17537, GSE14333 and GSE17536 datasets. **Figure. S3.** Reduced expression of MARCH8 in CRC tumor samples is associated with poor prognosis in patients with colorectal cancer (CRC). **A** Representative images of immunohistochemical analysis of the tumor samples obtained from the 85 patients with CRC. Based on the IHC analysis, the patients were divided into MARCH8-high and MARCH8-low groups. Scale bar: 100 μm. **B** MARCH8 mRNA levels in 25 CRC tumor and 25 paired normal tissue samples, which were obtained from the 85 patients with CRC. **C** Five-year survival rates of the 34 patients in the MARCH8-high group and the 51 patients in the MARCH8 low group. **Figure S4.** MARCH8 protein levels are negatively correlated with that of HK2 in human CRC tumor tissues. **A** Protein levels of MARCH8 and HK2 in 16 CRC tumor tissues and 8 normal colon tissues. **B** Densitometrial analysis on the ratios of MARCH8 and HK2 band. **C** Pearson correlation analysis of the correlation between HK2 protein levels and MARCH8. **Table S1. **Demographic and pathological data of the 85 patients with CRC, who were included in this study. **Table S2.** Short hairpin sequences of MARCH8 in this study. **Table S3.** Primer sequences used in this study. **Table S4.** Correlation of MARCH8 expression in colorectal cancer tissues with different clinicopathological features (n = 85).

**Additional file 2: Table S5.** Correlation of MARCH1-11 expression in colorectal cancer tissues with different clinicopathological features in TCGA COAD.

**Additional file 3: Table S6.** Correlation of MARCH8 expression in colorectal cancer tissues with different clinicopathological features in GSE12945.

## Data Availability

The data of this study are available from the corresponding authors for reasonable requests.

## Abbreviations

2-DG: 2-Deoxyglucose
CCK-8: Cell Counting Kit-8
CRC: Colorectal cancer
ECAR: Extracellular acidification rate
ELC: Enhanced chemiluminescence
GLUT1: Glucose transporter 1
GLUT4: Glucose transporter 4
HK2: Hexokinase 2
LDHA: Lactate dehydrogenase A
MARCH: Membrane associated RING-CH proteins
oeMAR: MARCH8 overexpression
MUT: Mutant
OCR: Oxygen consumption rate
OXPHOS: Oxidative phosphorylation
PKM2: Pyruvate kinase M2
shMARs: Short hairpin RNAs
UCP2: Uncoupling protein 2
WT: Wide type
