# Supplementary material for: Membrane-associated RING-CH protein (MARCH8) is a novel glycolysis repressor targeted by miR-32 in colorectal cancer
Source: J Transl Med. 2022 Sep 5;20:402. doi: 10.1186/s12967-022-03608-z (PMC9446774; doi:10.1186/s12967-022-03608-z)
Supplement: Supplementary file 1 — Additional file 1: Figure S1. The protein levels and Hazard Ratio of MARCH1-11 in TCGA-COAD, TCGA-READ, GSE12945 and GSE39582 datasets. A The protein levels of MARCH1-11 in TCGA-COAD, TCGA-READ, and GSE39582 datasets. B The forestplots of MARCH1-11 in TCGA-READ, GSE12945 and GSE39582 datasets. Figure S2. The expression levels and overall survival of MARCH8 in public datasets. A The expression levels of MARCH8 in TCGA-COAD, TCGA-READ, GSE41258 and GSE39582 datasets. B The overall survival of MARCH8 in TCGA-COAD, GSE41258, GSE12945, GSE39582, GSE17537, GSE14333 and GSE17536 datasets. Figure. S3. Reduced expression of MARCH8 in CRC tumor samples is associated with poor prognosis in patients with colorectal cancer (CRC). A Representative images of immunohistochemical analysis of the tumor samples obtained from the 85 patients with CRC. Based on the IHC analysis, the patients were divided into MARCH8-high and MARCH8-low groups. Scale bar: 100 μm. B MARCH8 mRNA levels in 25 CRC tumor and 25 paired normal tissue samples, which were obtained from the 85 patients with CRC. C Five-year survival rates of the 34 patients in the MARCH8-high group and the 51 patients in the MARCH8 low group. Figure S4. MARCH8 protein levels are negatively correlated with that of HK2 in human CRC tumor tissues. A Protein levels of MARCH8 and HK2 in 16 CRC tumor tissues and 8 normal colon tissues. B Densitometrial analysis on the ratios of MARCH8 and HK2 band. C Pearson correlation analysis of the correlation between HK2 protein levels and MARCH8. Table S1. Demographic and pathological data of the 85 patients with CRC, who were included in this study. Table S2. Short hairpin sequences of MARCH8 in this study. Table S3. Primer sequences used in this study. Table S4. Correlation of MARCH8 expression in colorectal cancer tissues with different clinicopathological features (n = 85). [file 12967_2022_3608_MOESM1_ESM.docx]

**Additional file**

**Figure**


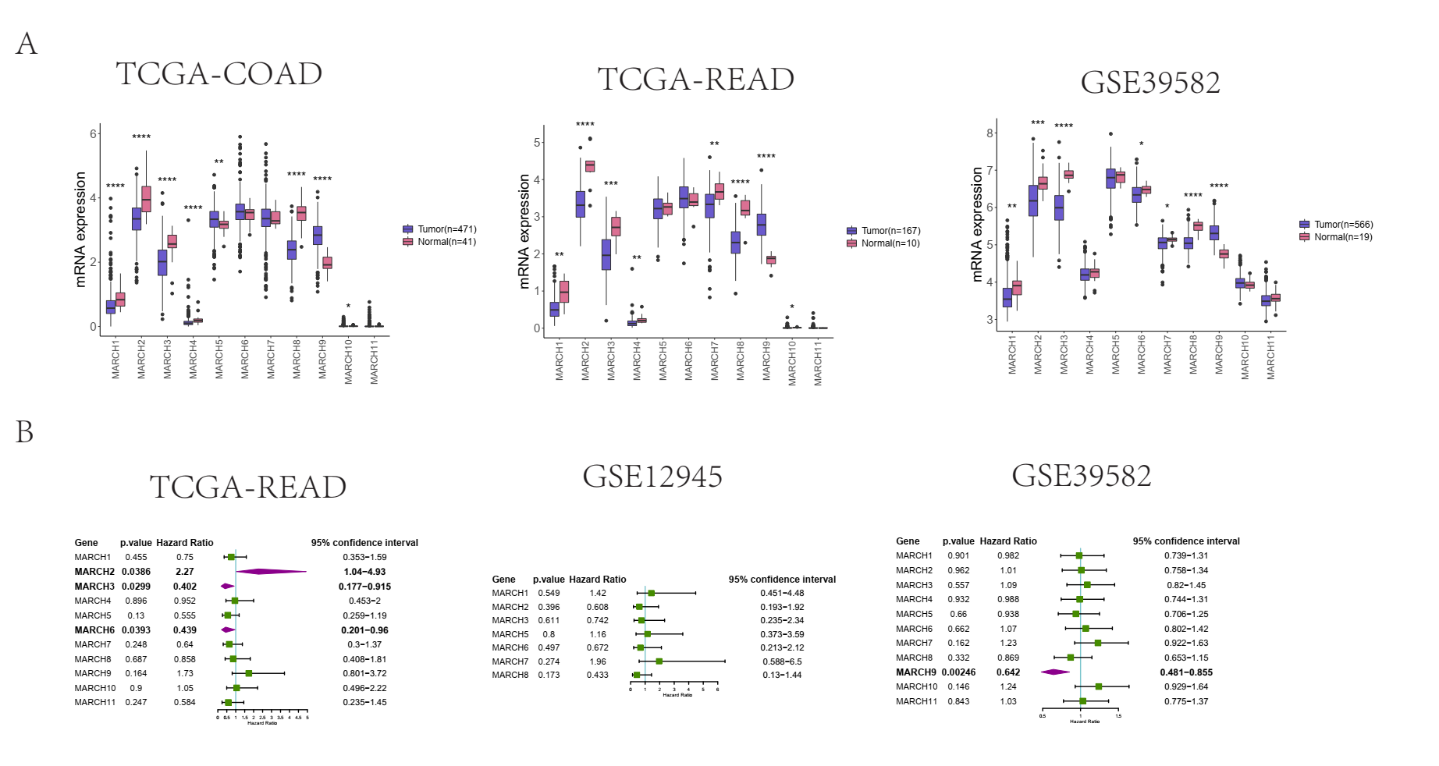
**Figure S1.** **The protein levels and Hazard Ratio of MARCH1-11 in TCGA-COAD, TCGA-READ, GSE12945 and GSE39582 datasets.**

(A) The protein levels of MARCH1-11 in TCGA-COAD, TCGA-READ, and GSE39582 datasets..

(B) The forestplots of MARCH1-11 in TCGA-READ, GSE12945 and GSE39582 datasets.


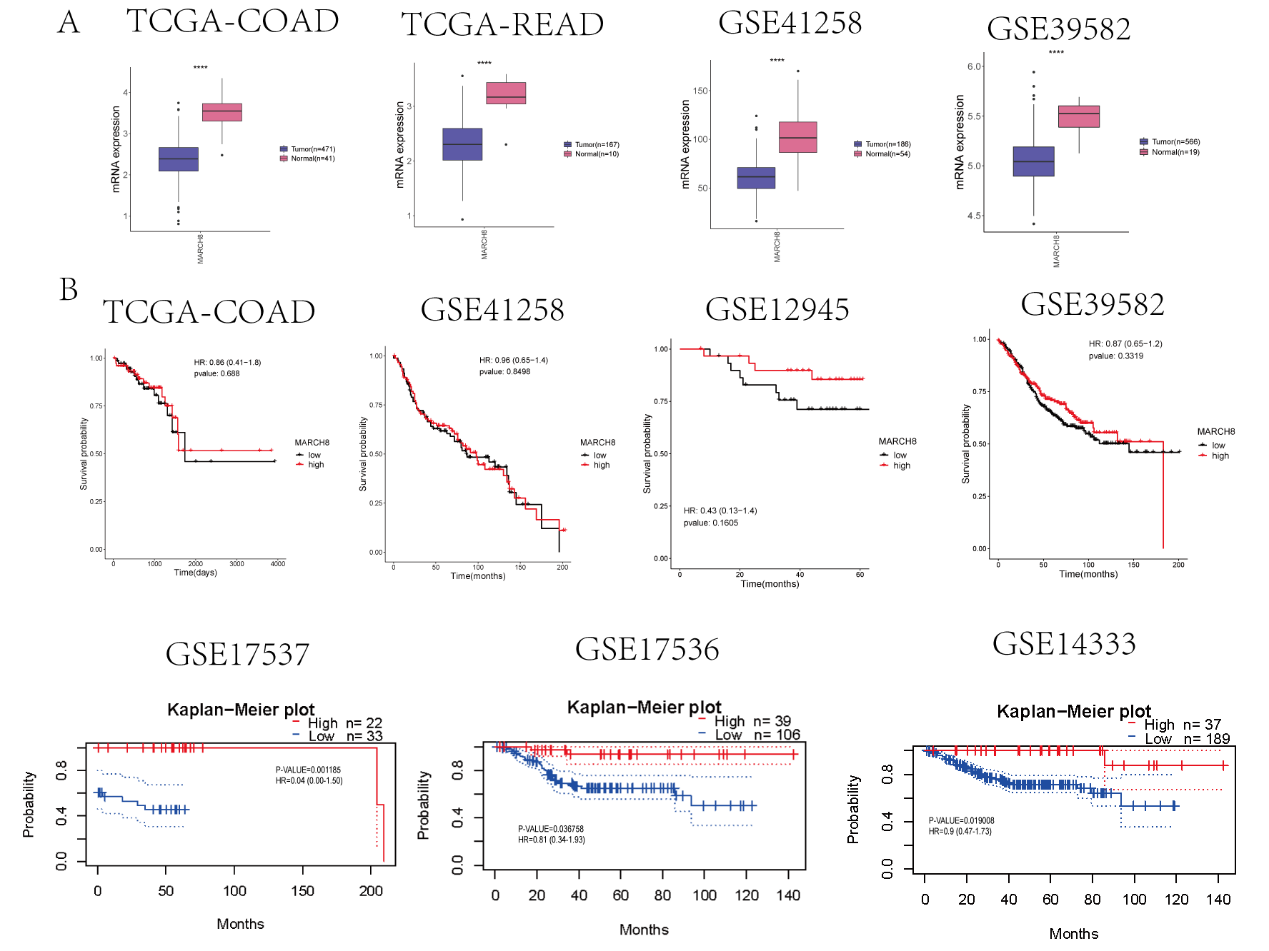


**Revised Figure S2 The expression levels and overall survival of MARCH8 in public datasets.**

(A) The expression levels of MARCH8 in TCGA-COAD, TCGA-READ, GSE41258 and GSE39582 datasets.

(B) The overall survival of MARCH8 in TCGA-COAD, GSE41258, GSE12945, GSE39582, [GSE17537](http://www.ncbi.nlm.nih.gov/geo/query/acc.cgi?acc=GSE17537), [GSE14333](http://www.ncbi.nlm.nih.gov/geo/query/acc.cgi?acc=GSE17537) and GSE17536 datasets.


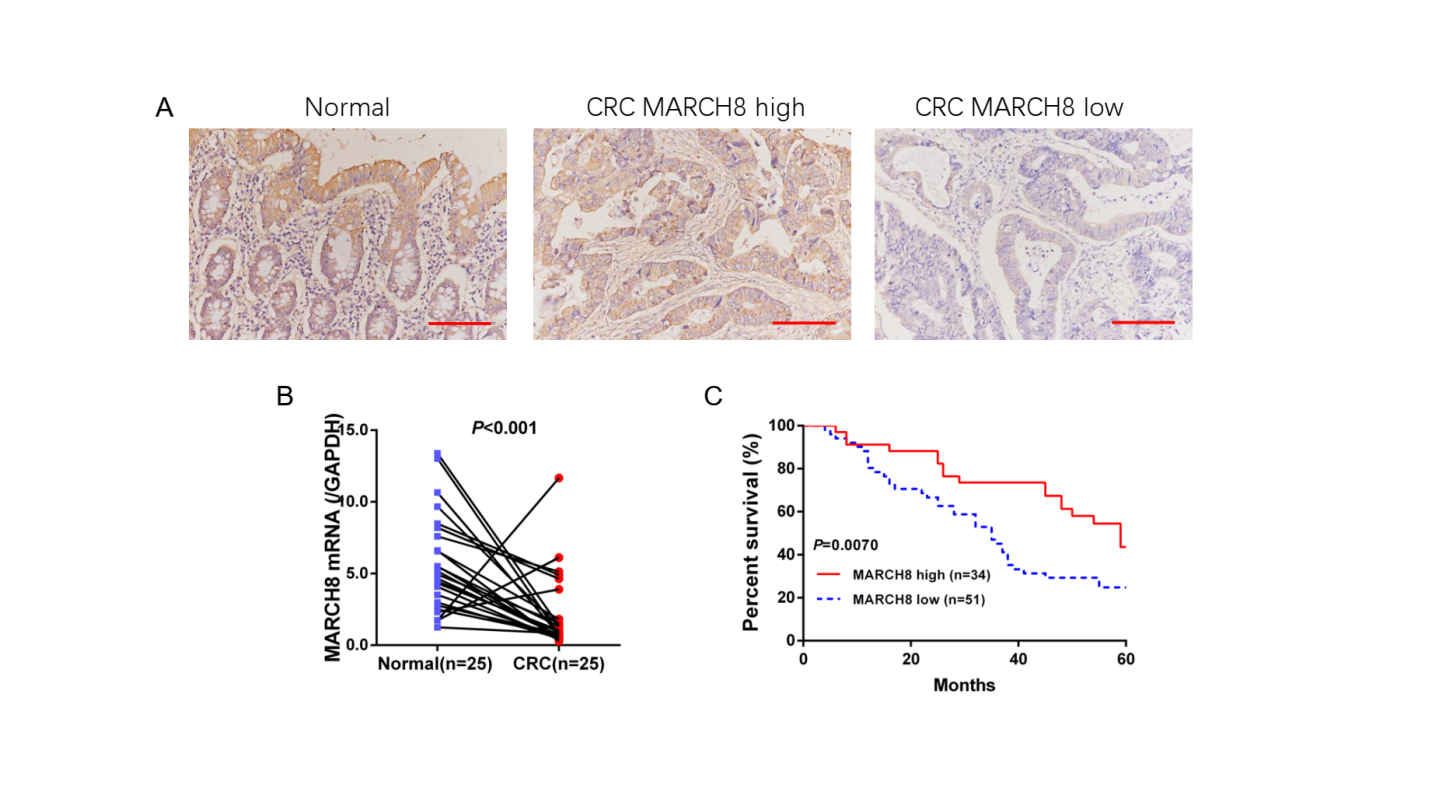


**Figure. S3 Reduced expression of MARCH8 in CRC tumor samples is associated with poor prognosis in patients with colorectal cancer (CRC).**

(A) Representative images of immunohistochemical analysis of the tumor samples obtained from the 85 patients with CRC. Based on the IHC analysis, the patients were divided into MARCH8-high and MARCH8-low groups. Scale bar: 100 μm.

(B) MARCH8 mRNA levels in 25 CRC tumor and 25 paired normal tissue samples, which were obtained from the 85 patients with CRC.

(C) Five-year survival rates of the 34 patients in the MARCH8-high group and the 51 patients in the MARCH8 low group.


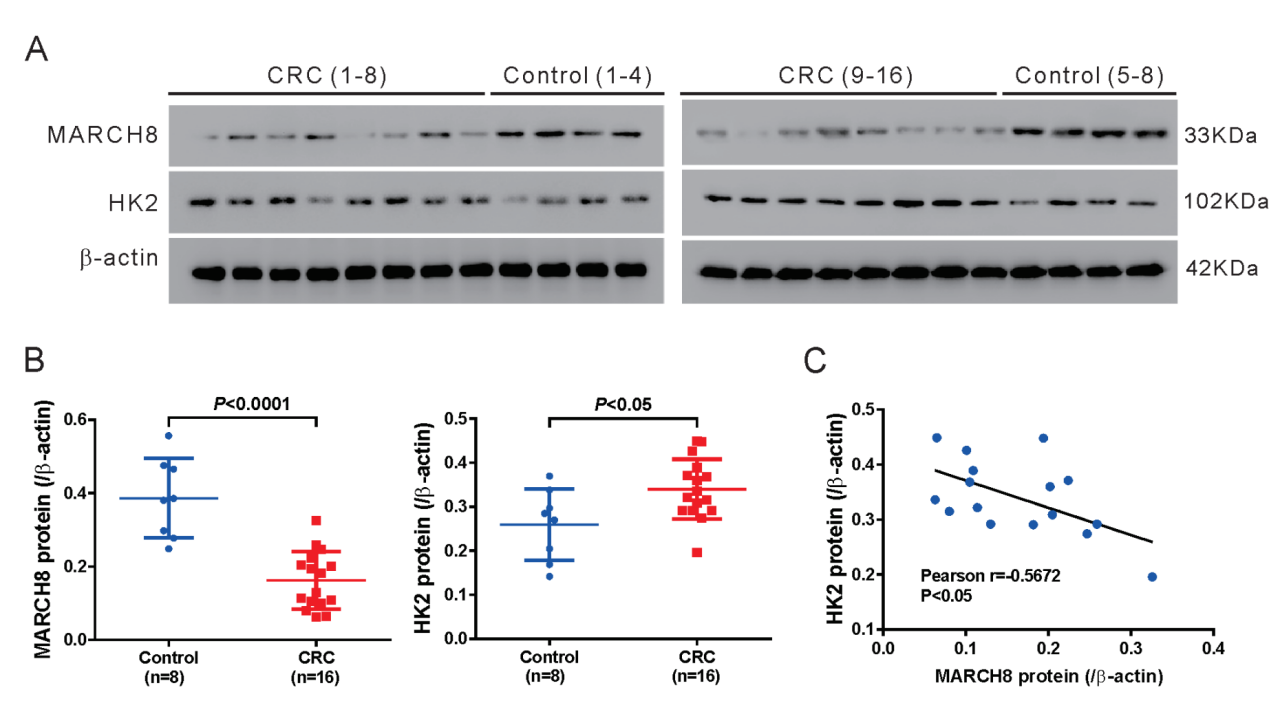


**Figure S4. MARCH8 protein levels are negatively correlated with that of HK2 in human CRC tumor tissues.**

(A) Protein levels of MARCH8 and HK2 in 16 CRC tumor tissues and 8 normal colon tissues.

(B) Densitometrial analysis on the ratios of MARCH8 and HK2 band.

(C) Pearson correlation analysis of the correlation between HK2 protein levels and MARCH8.

**Table**

**Table S1. Demographic and pathological data of the 85 patients with CRC, who were included in this study**

| **Characteristics** | **Cases** | **%** |
| --- | --- | --- |
| Gender |  |  |
| Male | 46 | 54.1 |
| Female | 39 | 45.9 |
| Age (years) |  |  |
| ≥65 | 48 | 56.5 |
| <65 | 37 | 43.5 |
| Tumor size (cm) |  |  |
| ≥5.0 | 46 | 54.1 |
| <5.0 | 39 | 45.9 |
| TNM stage |  |  |
| I/II | 38 | 44.7 |
| III | 47 | 55.3 |

**Table S2. Short hairpin sequences of MARCH8 in this study.**

| shRNAs | sequences |
| --- | --- |
| shMAR-1 | 5’-GCATGCCACTGCATCAGAT-3’ |
| shMAR-2 | 5’-GGAGAGGGAAGAACAGAAT-3’ |
| shMAR-3 | 5’-CCTTCTCTCGCACTTCTAT-3’ |

**Table S3. Primer sequences used in this study.**

| Genes | Forward | Reverse |
| --- | --- | --- |
| MARCH8 | 5’ GACTAAATTGGTGGTTGTG 3’ | 5’ TGATTTCTGCTCCAGTGTC 3’ |
| HK2 | 5’ GGCTCCAACGAGTTACCG 3’ | 5’ CACTTTGCCCATTTCAGG 3’ |
| GAPDH | 5’ GGATTGTCTGGCAGTAGCC 3’ | 5’ CACTTTGCCCATTTCAGG 3’ |

**Table S4. Correlation of MARCH8 expression in colorectal cancer tissues with different clinicopathological features (n = 85).**

| **Characteristic** | **MARCH8** | | ***P*-value** |
| --- | --- | --- | --- |
|  | **Low (n = 51)** | **High (n = 34)** |  |
| Gender |  |  | 0.5131 |
| Male | 28 | 18 |  |
| Female | 23 | 16 |  |
| Age (years) |  |  | 0.5052 |
| ≥65 | 27 | 21 |  |
| <65 | 24 | 13 |  |
| **Tumor size (cm)** |  |  | **0.0258*** |
| ≥5.0 | 33 | 13 |  |
| <5.0 | 18 | 21 |  |
| **Clinical stage** |  |  | **0.0037**** |
| I/II | 16 | 22 |  |
| III | 35 | 12 |  |
| **Survival status (at followed-up)** |  |  | **0.0124*** |
| Alive | 13 | 18 |  |
| Dead | 38 | 16 |  |

Clinicopathological features were assessed using the chi-square test. **P*<0.05, ***P*<0.01.
